# Clinical Cases

**Published:** 1888-02

**Authors:** Stuart Close

**Affiliations:** Brooklyn, N. Y.


### CLINICAL CASES.

STUART CLOSE, M. D., BROOKLYN, N. Y.

CASE I. *Intermittent Fever;* Mixed type.

Mrs. W., a coarse, ignorant German woman, aged thirty-five. Confined one week since. Being very poor, and having no one to help her she was compelled to get up on the sixth day, though very weak and sick. Has had chills and fever at intervals for several months, and almost constantly for the last month, increasing in severity.

*Status præsans;*—1. Chill and coldness predominate.

2. Cold all night in bed, with chill on moving.

3. Chills at eight A. M., eleven A. M., and three P. M.

4. Afternoon fever, beginning at about three o'clock, following the last chill.

5. During heat, pricking in skin, all over body, as from needles.

6. Great thirst, for large quantities, during all stages, can't get enough water.

7. Severe pains in the knees, aggravated by motion, bending the knees, and especially going down-stairs.

8. General aggravation from motion. "Feels pretty good when she keeps still, but miserable when tries to move about."

℞. Bryonia$^{200}$.

A powder was dissolved in water, and the patient directed to take a spoonful every three hours until a general sense of relief was experienced, and then to stop.

In four days she was cured. Two months later she reported that there had been no return of the fever.

From a hygienic point of view the surroundings of this woman were most unfavorable. Living in a miserable, filthy tenement, in a region notorious for its "malaria" and bad odors, confined, with no assistance but from a decrepit old woman, and compelled to get up from her bed on the sixth day and do her housework, one could hardly blame the homœopathic remedy for shirking its duty a little. But brave old Bryonia$^{200}$ ignored all such trifles, "rolled up his sleeves and went into it," and routed the intruder bag and baggage.

It illustrates the truth, that hygienic surroundings are not necessary to the successful use of our remedies.

CASE II. *Acute catarrhal inflammation of the middle ear.*

Miss B., aged nineteen. Very large, full figure, brown hair, blue eyes, fresh complexion.

A week ago, caught cold in some way, about the time the menses should have appeared. For several days she felt feverish, languid, with pain and stiffness all over the body. The menses delayed three days, then came on with a little more pain than usual. Her mother gave her Acon.$^{3}$ in frequent doses, the first day, which caused profuse perspiration, but did not materially modify the symptoms.

Yesterday she began to have earache, right side, gradually increasing in severity toward evening. The pain prevented her from sleeping during the night, in spite of treatment instituted and vigorously kept up by her mother.

This treatment consisted, first, of dropping Laudanum into the ear. Obtaining no relief from this, she resorted to the time-honored "onion treatment," which consists of roasting the onion, extracting the heart, and inserting it, while hot, into the painful ear.

In this way, during the thirty-six hours which had elapsed before I saw the case, she had used up a peck of onions, without relief. The mother, having "done *her* duty," sent for me. I found the following symptoms:

1. Face flushed bright red.
2. Right cheek and parotid region swollen, and very sensitive to touch.
3. Snapping and cracking in ear since four A. M.
4. Pain in paroxysms, excruciating, causing her to scream out and weep.
5. Pain is sharp, stitching, from within outward; also extends below ear along eustachian tube.
6. Paroxysms come suddenly, "like a flash of lightning," last about half a minute, and disappear as suddenly as they came.
7. During the paroxysm, she screams, throws herself about, *strikes at any one who comes near her.* Says she can't help it—feels as if she must strike somebody.

8. Pains across lower part of back and in uterine region, aggravated by turning in bed. ℞. Bell.$^{200}$.

She received, I think, two doses of a solution of the remedy, about twenty minutes apart. Relief was almost instant. In about two hours, all pain was gone, and she slept soundly all night. The ear was "stopped up" for a few days, but this soon passed away without further medicine.

---

